# Persistent Graves’ hyperthyroidism despite rapid negative conversion of thyroid-stimulating hormone-binding inhibitory immunoglobulin assay results: a case report

**DOI:** 10.1186/s13256-017-1214-6

**Published:** 2017-02-06

**Authors:** Nobumasa Ohara, Masanori Kaneko, Masaru Kitazawa, Yasuyuki Uemura, Shinichi Minagawa, Masashi Miyakoshi, Kenzo Kaneko, Kyuzi Kamoi

**Affiliations:** 1 Department of Endocrinology and Metabolism, Nagaoka Red Cross Hospital, 2-297-1 Senshu, Nagaoka, Niigata 940-2085 Japan; 2 Department of Endocrinology and Metabolism, Uonuma Institute of Community Medicine, Niigata University Medical and Dental Hospital, Niigata, Japan; 3 Tsutsui Medical Clinic, Niigata, Japan; 4 Department of Internal Medicine, Ojiya General Hospital, Niigata, Japan; 5 Center of Diabetes, Endocrinology and Metabolism, Joetsu General Hospital, Niigata, Japan

**Keywords:** Hyperthyroidism, Thyroid-stimulating hormone-binding inhibitory immunoglobulin, Graves’ disease, Thiamazole, Thyroid scintigraphy, Human leukocyte antigen

## Abstract

**Background:**

Graves’ disease is an autoimmune thyroid disorder characterized by hyperthyroidism, and patients exhibit thyroid-stimulating hormone receptor antibody. The major methods of measuring circulating thyroid-stimulating hormone receptor antibody include the thyroid-stimulating hormone-binding inhibitory immunoglobulin assays. Although the diagnostic accuracy of these assays has been improved, a minority of patients with Graves’ disease test negative even on second-generation and third-generation thyroid-stimulating hormone-binding inhibitory immunoglobulins. We report a rare case of a thyroid-stimulating hormone-binding inhibitory immunoglobulin-positive patient with Graves’ disease who showed rapid lowering of thyroid-stimulating hormone-binding inhibitory immunoglobulin levels following administration of the anti-thyroid drug thiamazole, but still experienced Graves’ hyperthyroidism.

**Case presentation:**

A 45-year-old Japanese man presented with severe hyperthyroidism (serum free triiodothyronine >25.0 pg/mL; reference range 1.7 to 3.7 pg/mL) and tested weakly positive for thyroid-stimulating hormone-binding inhibitory immunoglobulins on second-generation tests (2.1 IU/L; reference range <1.0 IU/L). Within 9 months of treatment with oral thiamazole (30 mg/day), his thyroid-stimulating hormone-binding inhibitory immunoglobulin titers had normalized, but he experienced sustained hyperthyroidism for more than 8 years, requiring 15 mg/day of thiamazole to correct. During that period, he tested negative on all first-generation, second-generation, and third-generation thyroid-stimulating hormone-binding inhibitory immunoglobulin assays, but thyroid scintigraphy revealed diffuse and increased uptake, and thyroid ultrasound and color flow Doppler imaging showed typical findings of Graves’ hyperthyroidism.

**Conclusions:**

The possible explanations for serial changes in the thyroid-stimulating hormone-binding inhibitory immunoglobulin results in our patient include the presence of thyroid-stimulating hormone receptor antibody, which is bioactive but less reactive on thyroid-stimulating hormone-binding inhibitory immunoglobulin assays, or the effect of reduced levels of circulating thyroid-stimulating hormone receptor antibody upon improvement of thyroid autoimmunity with thiamazole treatment. Physicians should keep in mind that patients with Graves’ disease may show thyroid-stimulating hormone-binding inhibitory immunoglobulin assay results that do not reflect the severity of Graves’ disease or indicate the outcome of the disease, and that active Graves’ disease may persist even after negative results on thyroid-stimulating hormone-binding inhibitory immunoglobulin assays. Timely performance of thyroid function tests in combination with sensitive imaging tests, including thyroid ultrasound and scintigraphy, are necessary to evaluate the severity of Graves’ disease and treatment efficacy.

## Background

Graves’ disease (GD) is an autoimmune thyroid disorder characterized by hyperthyroidism (increased thyroid hormone synthesis and secretion) that is often associated with goiter and exophthalmos [1]. Patients with GD exhibit circulating immunoglobulins, particularly thyroid-stimulating hormone (TSH) receptor antibody (TRAb), that bind to and stimulate the TSH receptors and result in sustained overactivity of the thyroid gland.

The major methods of assessing TRAb to diagnose GD include measurement of the thyroid-stimulating antibody (TSAb) index using a functional bioassay and determination of TSH-binding inhibitory immunoglobulin (TBII) levels by a radioligand receptor assay [2]. The diagnostic accuracy of TBII has been improved over three generations of laboratory methods [3], and TBII titers usually reflect the degree of hyperthyroidism and can predict potential remission or relapse [4, 5]. However, a few patients with active GD show negative test results even on second-generation and third-generation TBII assays [6, 7]. Patients with TBII-negative GD may have mild hyperthyroidism, smaller goiters, weak TSAb activity, minimal radioactive iodine uptake, and a better prognosis in relation to the effect of anti-thyroid drug treatments [8, 9]. One unusual case report described a patient with GD who initially tested negative for TSAb but positive for second-generation TBII; the patient rapidly became negative for second-generation TBIIs following treatment with the anti-thyroid drug Thiamazole (methimazole; MMI) [10].

We report a case involving a patient with GD who had severe hyperthyroidism and was TSAb-negative and TBII-positive on second-generation tests. Following administration of MMI, his TBII results rapidly became negative, although he continued to experience Graves’ hyperthyroidism for a prolonged period.

## Case presentation

A 45-year-old Japanese man was referred to our hospital in March 2007 because of thyrotoxicosis. His family history was unremarkable, but he had a medical history of thyrotoxicosis that was treated with oral MMI at a local hospital from 23 to 43 years of age, at which time he discontinued the therapy based on his own judgment. He had smoked 20 cigarettes per day since he was 20-years old, had taken no medication (except for MMI), would drink a glass of beer on a social basis, and ingested an adequate amount of iodine with the traditional Japanese diet. He visited his primary care doctor for the first time in the previous 2 years because of months of fatigue, palpitations, and finger tremors and was diagnosed with thyrotoxicosis. He was subsequently referred to our hospital.

A physical examination revealed that he was 161 cm tall, weighed 51 kg, had a body temperature of 36.8 °C, and had a blood pressure of 143/75 mmHg. He did not exhibit exophthalmos or skin eruption but presented with a soft and mild goiter without pain, moist skin, and bilateral finger tremors. In addition, thrill and vascular bruit were audible on his goiter. No heart murmurs, chest rales, or peripheral edema were detected, and an electrocardiogram revealed sinus tachycardia with a heart rate of 108 beats per minute. His laboratory data revealed a normal complete blood count, high serum alkaline phosphatase level, and severe hyperthyroidism: free triiodothyronine (FT_3_) >25.0 pg/mL and free thyroxine (FT_4_) 7.90 ng/dL (Table 1). His TSAb test (Yamasa Corporation; Chiba, Japan) was negative, but his second-generation TBII (Yamasa Corporation), thyroid peroxidase antibody (TPOAb)-radioimmunoassay (RIA; Cosmic Corporation; Tokyo, Japan), and thyroglobulin antibody (TgAb)-RIA (Cosmic Corporation) tests were positive. Thyroid ultrasonography showed a diffusely enlarged thyroid gland in the absence of a tumor lesion, and color flow Doppler detected increased blood flow. Thus, he was considered to have Graves’ hyperthyroidism, and he began treatment with oral MMI (30 mg/day). In addition, he was instructed to discontinue smoking cigarettes.

**Table 1:** Laboratory findings in March 2007

Hematology		
Red blood cells	489×10^4^/μL	(427–571)
Hemoglobin	14.2 g/dL	(12.4–17.2)
Hematocrit	42.1%	(38.7–50.3)
White blood cells	5300/μL	(4000–9000)
Platelets	14.8×10^4^/μL	(12.0–30.0)
Chemistry		
Total protein	6.7 g/dL	(6.7–8.3)
Albumin	3.9 g/dL	(3.8–5.3)
Aspartate aminotransferase	24 IU/L	(13–33)
Alanine aminotransferase	29 IU/L	(8–42)
Urea nitrogen	13.8 mg/dL	(8.0–22.0)
Creatinine	0.44 mg/dL	(0.40–0.70)
Sodium	143 mmol/L	(137–147)
Potassium	4.3 mmol/L	(3.5–4.7)
Chloride	107 mmol/L	(98–108)
C-reactive protein	0.08 mg/dL	(<0.30)
Casual plasma glucose	6.1 mmol/L	(3.9–7.8)
Total cholesterol	135 mg/dL	(130–220)
Triglycerides	76 mg/dL	(50–130)
Thyroid-stimulating hormone	<0.01 μU/mL	(0.30–4.30)
Free triiodothyronine	>25.0 pg/mL	(1.71–3.71)
Free thyroxine	7.90 ng/dL	(0.70–1.48)
Second-generation TBII	2.1 IU/L	(<1.0)
Thyroid-stimulating antibody	156%	(0–180)
Thyroid peroxidase antibody	84.3 U/mL	(<0.3)
Thyroglobulin antibody	185 U/mL	(<0.3)
Anti-nuclear antibody	<40 titer	(<40)

The reference range for each parameter is shown in parentheses. *TBII* thyroid-stimulating hormone-binding inhibitory immunoglobulin

**Fig. 1:**
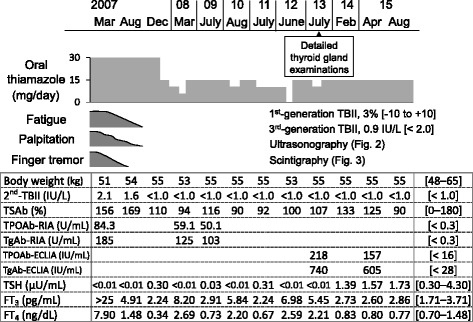
Clinical course of the patient. The reference ranges for body weight, serum levels of free triiodothyronine, free thyroxine, and thyroid-stimulating hormone; and titers from the first-generation, second-generation, and third-generation thyroid-stimulating hormone-binding inhibitory immunoglobulin, thyroid-stimulating antibody, thyroid peroxidase antibody-radioimmunoassay, thyroglobulin antibody-radioimmunoassay, thyroid peroxidase antibody-electrochemiluminescence immunoassay, and thyroglobulin antibody-electrochemiluminescence immunoassay tests are shown in *square brackets*. The reference range for body weight (kg) was calculated as follows: (body height in m^2^ × 18.5) to (body height in m^2^ × 25). *ECLIA* electrochemiluminescence immunoassay, *FT*_*3*_ free triiodothyronine, *FT*_*4*_ free thyroxine, *RIA* radioimmunoassay, *TBII* thyroid-stimulating hormone-binding inhibitory immunoglobulin, *TgAb* thyroglobulin antibody, *TPOAb* thyroid peroxidase antibody, *TSAb* thyroid-stimulating antibody, *TSH* thyroid-stimulating hormone

After 3 months of treatment with oral MMI, his serum thyroid hormone levels decreased to almost within the normal range, and he experienced improvements in his palpitations, sweating, and finger tremors and gained 4 kg of body weight during this period. In December 2007, he had a normal serum FT_3_ level (2.24 pg/mL) with a detectable TSH level, and his second-generation TBII titer had normalized (Fig. 1); thus, his dose of MMI was titrated to <10 mg/day. However, he exhibited persistent hyperthyroidism and continued oral MMI treatment at 10 to 15 mg/day.

He discontinued the oral MMI treatment of his own accord in April 2012 and then revisited our hospital in June 2012 due to fatigue and palpitations. Laboratory findings revealed hyperthyroidism (FT_3_ 6.98 pg/mL, FT_4_ 2.59 ng/dL; Fig. 1). He resumed medication with oral MMI (15 mg/day); his thyrotoxicosis symptoms had resolved and his serum FT_3_ and FT_4_ levels had normalized within 3 months.

**Fig. 2:**
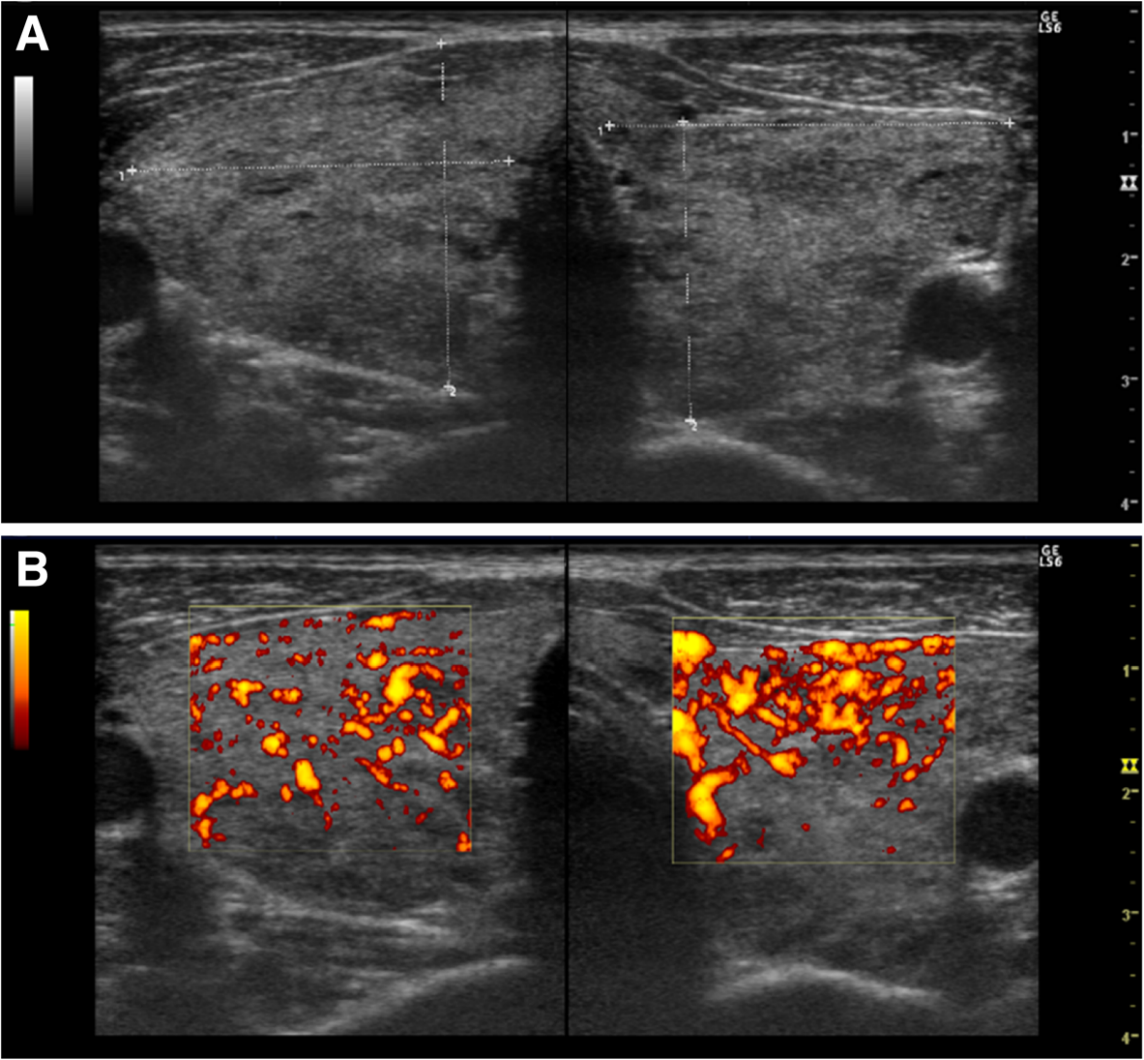
Ultrasonography of the thyroid gland (July 2013). **a** Ultrasonography showing heterogeneous and low echogenicity in a diffusely enlarged thyroid gland. No tumor was detected in the thyroid gland. **b** Color flow Doppler imaging revealing increased blood flow

**Fig. 3:**
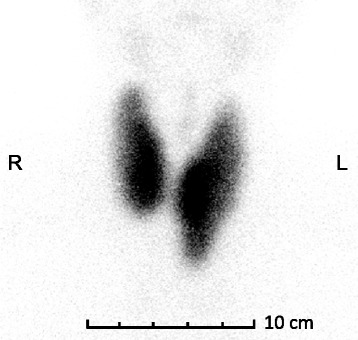
Thyroid scintigraphy (July 2013). Technetium-99 m sodium pertechnetate thyroid scan (anterior view) showing a diffuse accumulation in the thyroid gland with an increased thyroid uptake rate of 14.2% (reference range 0.5 to 4.0%)

A detailed examination of his unresolved thyrotoxicosis was performed in July 2013. At this time, he presented with a soft goiter with audible bruit but did not exhibit exophthalmos or skin eruption. A blood chemistry analysis showed slightly high serum levels of FT_3_ (5.45 pg/mL) and FT_4_ (2.21 ng/dL) under treatment with oral MMI (10 mg/day). He tested negative on TSAb (107%) and first-generation (3%; reference range –10 to +10%; Cosmic Corporation), second-generation (<1.0 IU/L), and third-generation (0.9 IU/L; reference range <2.0 IU/L; Roche Diagnostics K.K.; Tokyo, Japan) TBII assays. He showed positive test results on a TPOAb-electrochemiluminescence immunoassay (ECLIA; TPOAb titer 218 IU/mL, reference range <16.0 IU/mL; Roche Diagnostics K.K.) and a TgAb-ECLIA (TgAb titer 740 IU/mL, reference range <28.0 IU/mL; Roche Diagnostics K.K.). A thyroid ultrasonography showed heterogeneous and reduced echogenicity in a diffusely enlarged thyroid gland without a mass lesion, and color flow Doppler detected increased blood flow (Fig. 2). Technetium-99 m thyroid scintigraphy showed diffuse and elevated uptake (Fig. 3). These findings indicated persistent Graves’ hyperthyroidism [1]. Human leukocyte antigen (HLA) typing revealed the presence of A*24:02:01/31:01:02, B*40:02:01/51:01:01, and C*03:04:01/14:02:01 class I genes and DRB1*04:10:01/12:01:01, DQB1*03:03:02/04:02:01, DQA1*03:02/03:03, and DPB1*05:01:01/(−) class II genes.

In terms of the long-term management of his hyperthyroidism, he was informed of various treatment options, such as radioiodine therapy and thyroidectomy, but he expressed his hope to continue with the anti-thyroid medication. Because the MMI treatment had previously appeared to effectively control his hyperthyroidism with no obvious side effects, he continued oral MMI treatment at 15 mg/day (Fig. 1). Accordingly, his peripheral thyroid hormone levels were maintained within approximately normal reference ranges during the course of the MMI treatment.

## Discussion

Our Japanese patient had a goiter and severe thyrotoxicosis; he tested negative for TSAb but weakly positive on a second-generation TBII assay. Within 9 months of treatment with oral MMI (30 mg/day), his TBII titers had normalized; however, he continued to experience sustained thyrotoxicosis, which required 15 mg/day of MMI treatment to correct, for more than 8 years. During that period, he tested negative on first-generation, second-generation, and third-generation TBII assays (Fig. 1). Thyroid scintigraphy showed diffuse and increased uptake of technetium-99 m (Fig. 3) and indicated persistent hyperthyroidism.

The common causes of hyperthyroidism include GD as well as toxic multinodular goiter or solitary toxic nodules, which result from thyroid autonomy due to activating mutations of TSH receptor gene [11]. Hyperthyroidism is also rarely caused by TSH-producing pituitary tumors, congenital disorders, and administration of drugs, such as iodine or amiodarone. In the present case, our patient showed transient but significant elevation of second-generation TBII titers before administration of MMI. Thyroid ultrasound and color flow Doppler, performed before and after normalization of TBII titers, showed hypoechoic heterogeneous echogenicity and hypervascularity in a diffusely enlarged thyroid gland without a tumor lesion (Fig. 2). These findings indicate that he exhibited persistent Graves’ hyperthyroidism.

The reasons for the negative TBII findings in the presence of persistent hyperthyroidism in our patient remain unclear. However, TRAb is known to be heterogeneous in both molecular structure and biological activity with a propensity to change during the course of GD [5]. Anti-thyroid drug treatment usually reduces TBII titers [4] possibly by improving thyroid autoimmunity [12, 13]. In addition, even second-generation or third-generation TBII assays may not be sensitive enough to detect circulating TRAb in patients with TBII-negative GD, or intrathyroidal TRAb production may not spill over to the circulation. In the present case (Fig. 1), our patient showed a slight elevation of TBII titers in the presence of severe hyperthyroidism before MMI treatment. In addition, although his TSAb index remained within the reference range throughout the course of his disease, his TSAb index decreased in parallel with the conversion of TBII results to negative after MMI treatment, as was observed in a previous case report [10]. These findings suggest that our patient probably exhibited circulating TRAb that were bioactive enough to cause hyperthyroidism but less reactive on TBII or TSAb assays. In addition, MMI treatment decreased his TBII titers, possibly by decreasing the reactivity of TRAb to TBII and TSAb assays or by reducing the levels of circulating TRAb.

Both TPOAb and TgAb are circulating autoantibodies that recognize thyroid tissue [14]. Patients with GD usually test positive for TPOAb and TgAb, and treatment of Graves’ hyperthyroidism with anti-thyroid drugs decreases titers of these autoantibodies [15]. Our patient tested positive for TPOAb and TgAb, and the titers decreased during treatment with MMI. Although these thyroid autoantibodies do not have a specific role in the diagnosis of GD [1], the positivity for and decreasing titers of TPOAb and TgAb in our patient support the diagnosis of GD and suggest an improvement in his thyroid autoimmunity.

The pathogenesis of GD is multifactorial and involves a complex interaction between environmental and genetic factors. Substantial ethnic differences in GD genetic predispositions exist between white and Japanese populations [16, 17]. In addition, there may be differences in genetic background between TBII-positive and TBII-negative patients with GD. An interesting study of Japanese patients with GD demonstrated that HLA-DPB1*05:01 is associated with typical TBII-positive GD, whereas HLA-DPB1*02:02 is frequent in patients who have negative TBII assay results at their first visit and throughout their entire clinical course [18]. In the present case, HLA-DPB1*05:01 homozygosity might have been involved in the development of his GD, and this may have manifested as a full-blown goiter and long-standing severe hyperthyroidism despite the rapid negative conversion of TBII assay results.

## Conclusions

We described a rare case of a TBII-positive patient with GD who showed rapid lowering of TBII levels following administration of MMI, but still experienced hyperthyroidism for more than 8 years. Possible explanations for the serial changes in the TBII assay results in our patient include the presence of TRAb that is bioactive but less reactive on TBII assays, or reduced levels of circulating TRAb upon improvement of thyroid autoimmunity by MMI treatment. This case highlights the need for physicians to consider that patients with GD may show TBII or TSAb test results that do not reflect the severity of GD or predict the outcome of the disease, and that active GD may persist even after negative conversion of both TBII and TSAb results. The timely performance of thyroid function tests in combination with sensitive imaging tests, including thyroid ultrasound and scintigraphy, are necessary to evaluate the severity and treatment efficacy in GD.

## Abbreviations

ECLIA: Electrochemiluminescence immunoassay
FT_3_: Free triiodothyronine
FT_4_: Free thyroxine
GD: Graves’ disease
HLA: Human leukocyte antigen
MMI: Methimazole
RIA: Radioimmunoassay
TBII: Thyroid-stimulating hormone-binding inhibitory immunoglobulin
TgAb: Thyroglobulin antibody
TPOAb: Thyroid peroxidase antibody
TRAb: Thyroid-stimulating hormone receptor antibody
TSAb: Thyroid-stimulating antibody
TSH: Thyroid-stimulating hormone
